# Revisiting the Quantum Brain Hypothesis: Toward Quantum (Neuro)biology?

**DOI:** 10.3389/fnmol.2017.00366

**Published:** 2017-11-07

**Authors:** Peter Jedlicka

**Affiliations:** Institute of Clinical Neuroanatomy, Neuroscience Center, Goethe University Frankfurt, Frankfurt, Germany

**Keywords:** quantum biology, quantum neurobiology, complexity theory, non-linear dynamics, neuronal avalanches, criticality

## Abstract

The nervous system is a non-linear dynamical complex system with many feedback loops. A conventional wisdom is that in the brain the quantum fluctuations are self-averaging and thus functionally negligible. However, this intuition might be misleading in the case of non-linear complex systems. Because of an extreme sensitivity to initial conditions, in complex systems the microscopic fluctuations may be amplified and thereby affect the system’s behavior. In this way quantum dynamics might influence neuronal computations. Accumulating evidence in non-neuronal systems indicates that biological evolution is able to exploit quantum stochasticity. The recent rise of quantum biology as an emerging field at the border between quantum physics and the life sciences suggests that quantum events could play a non-trivial role also in neuronal cells. Direct experimental evidence for this is still missing but future research should address the possibility that quantum events contribute to an extremely high complexity, variability and computational power of neuronal dynamics.

## Introduction

The view of the nervous system as a linear, computer-like machine performing classical, deterministic input-output or stimulus-response computations is still very popular in neuroscience. However, this view is challenged by experimental findings and theoretical analyses indicating that the nervous system is a non-linear dynamical complex system (Deco et al., 2008; Singer, 2009; Tognoli and Scott Kelso, 2014; Wolf et al., 2014) exhibiting highly stochastic activity (Deco et al., 2009). There is a need for a *“paradigm shift from behaviorist stimulus-response concepts toward notions of predictive coding in self-organizing recurrent networks with high dimensional dynamics”* (Singer, 2015). Neural networks consist of nerve cells that are linked by many reciprocal connections (Markov and Kennedy, 2013; Singer, 2013) and are capable of non-linear computations (London and Häusser, 2005). Neuronal networks with non-linear neurons and densely connected feedback loops can generate dynamics that is more complex, variable and rich than expected (Deco and Jirsa, 2012; Singer, 2013; Singer and Lazar, 2016).

The complexity of neuronal dynamics and its capability of fast parallel processing of information is thought to arise from powerful but purely classical computing strategies. The high diversity of neural network dynamics is considered to emerge primarily from non-linearities in the behavior of network nodes and from high variability in the strength and conduction delays of network connections. A conventional wisdom is that in macroscopic objects such as our brain the quantum fluctuations are self-averaging and thus cannot contribute to its rich dynamics. Indeed, it is very likely that the nervous system cannot display macroscopic quantum (classically impossible) behaviors such as quantum entanglement, superposition or tunneling (Koch and Hepp, 2006). Therefore, the prevailing view has been that quantum processes are irrelevant for the brain function. However, in contrast to quantum brain proposals based on implausible quantum mechanisms, there is an alternative, more realistic and subtle way in which quantum events might influence the brain activity and increase its computational power and information coding abilities. Part of this article has been published as a part of a book chapter and is adapted here with permission (Jedlicka, 2014).

## Two Arguments Against the Quantum Brain Hypothesis

There are two main arguments, which are usually raised against the quantum brain hypothesis:

(1) Neuronal signaling molecules, neurons and neural networks are **too large** for quantum phenomena to play a significant role in their functioning. The conventional wisdom is that all *quantum events* are *averaging out*, so that fluctuations among quantum particles are not important. As expressed by Daniel Dennett:

“Most biologists think that quantum effects all just cancel out in the brain, that there’s no reason to think they’re harnessed in any way. Of course they’re there; quantum effects are there in your car, your watch, and your computer. But most things — most macroscopic objects — are, as it were, oblivious to quantum effects. They don’t amplify them; they don’t hinge on them” (Penrose and Dennett, 1995).

Christoph Koch and Klaus Hepp, too, identify the large size of neuronal objects and the huge number of particles involved in neuronal signaling as one of the critical weak points of quantum brain hypothesis:

“Although brains obey quantum mechanics, they do not seem to exploit any of its special features. Molecular machines, such as the light-amplifying components of photoreceptors, pre- and post-synaptic receptors and the voltage- and ligand-gated channel proteins that span cellular membranes and underpin neuronal excitability, are so large that they can be treated as classical objects. … Two key biophysical operations underlie information processing in the brain: chemical transmission across the synaptic cleft, and the generation of action potentials. These both involve 1000s of ions and neurotransmitter molecules, coupled by diffusion or by the membrane potential that extends across 10s of micrometers. Both processes will destroy any coherent quantum states. Thus, spiking neurons can only receive and send classical, rather than quantum, information. It follows that a neuron either spikes at a particular point in time or it does not, but is not in a superposition of spike and non-spike states” (Koch and Hepp, 2006).

(2) The second important criticism is that the *interaction* of neuronal molecules, neurons, or neuronal networks with their noisy, wet and warm *environment* will *destroy* any *non-trivial quantum states* such as superpositions or entanglements. If this were true, only trivial quantum effects could be present in the nervous system. But what is the difference between trivial and non-trivial quantum effects? Trivial quantum effects provide the basis for the structure and chemical properties of molecules and they are ubiquitous (also in cars and watches). Hence, trivial quantum effects are crucial for the basic biochemistry of neuronal molecules, but when considering the neuronal function, these trivial quantum features can be ignored and the molecules important for neuronal signaling can, allegedly, be treated as essentially classical. All interesting coherent quantum states which are necessary for any non-trivial quantum computation (Davies, 2004) can, allegedly, exist only in well-isolated quantum states and are rapidly destroyed by the environment. Since the brain is “a 300-degrees Kelvin tissue strongly coupled to its environment” (Koch and Hepp, 2006), decoherence will prevail and no neuronal quantum computation will be possible. Because of the extremely high speed of environment-induced decoherence, the brain “should be thought of as a classical rather than quantum system” (Tegmark, 2000).

## Two Possibilities of the Impact by Quantum Events

The two arguments against the hypothesis that quantum dynamics play a non-trivial role in the nervous system seem convincing and are accepted by most scientists. However, a growing body of empirical evidence indicates that the second argument is false and that the first argument is also very likely false. Exciting recent research shows that *non-trivial quantum effects* are present *in biological systems* – and not just in spite of, but sometimes because of, the interaction with the noisy and warm environment. Furthermore, because the brain is a *complex non-linear system* with high sensitivity to small fluctuations, it is likely that it can *amplify* microscopic *quantum effects*. Specifically, there are two alternative but interrelated ways in which quantum events may influence the activity of the brain (Satinover, 2001, see also Jedlicka, 2005, 2009): (1) Non-trivial quantum effects can speed up the computational processes in living organisms at the microscopic level. (2) Non-linear chaotic dynamics can amplify lowest-level quantum fluctuations upward, modulating even larger-scale mesoscopic and maybe also macroscopic neuronal activity.

What is the experimental evidence for these two claims?

(1) Contrary to expectations, non-trivial quantum processes have been observed in living systems (Arndt et al., 2009; Brookes, 2017). Experiments provide evidence for unexpectedly long-lasting quantum coherence in the electron transfer which is involved in photosynthesis (Engel et al., 2007; Lee et al., 2007; Collini et al., 2010; Panitchayangkoon et al., 2010, 2011; Sarovar et al., 2010). This quantum-mechanical process is thought to improve the efficiency of energy transfer in photopigment molecules (Panitchayangkoon et al., 2010). The pigment molecules seem to implement an efficient quantum algorithm to find the fastest route for the light-induced excitation of electrons (Sension, 2007). Quantum coherence (Chenu and Scholes, 2015) has been found in photosynthetic bacteria as well as in marine algae. This suggests that evolution has been able to select and exploit quantum-mechanical features for fast and efficient computation in two evolutionary distinct organisms (but see also Duan et al., 2017). Another example of quantum dynamics in living systems has been found in photoreceptors, which are important for vision. Photoreceptor cells of the retina contain a protein called rhodopsin. Experiments using high-resolution spectroscopic and nuclear-magnetic resonance techniques revealed coherent quantum waves in the rhodopsin molecule (Wang et al., 1994; Loewenstein, 2013b). As summarized by Werner Loewenstein: *“Quantum mechanics, not classical mechanics, rules the roost at this sensory outpost of the brain*” (Loewenstein, 2013b).

Quantum effects have been described also in the olfactory system. Electron tunneling has been suggested to play an important role in the detection of odorants by olfactory receptors (Huelga and Plenio, 2013). Avian magnetoreception is yet another example of potentially beneficial quantum effects in biology. Long-lived quantum entanglements in the cryptochromes of the retina seem to support the sensitivity of a bird’s eye to magnetic fields (Arndt et al., 2009; Ball, 2011; Huelga and Plenio, 2013). Recent simulations show that quantum mechanical coherences in cryptochrome models can account for the precision of the avian magnetic compass (Hiscock et al., 2016). In addition, quantum tunneling has been observed in other biomolecules, such as enzymes (Klinman and Kohen, 2013) or motor proteins (Hunter, 2006). Most importantly, contrary to the long-held view, under some conditions, the strong *coupling to the noisy and warm environment is able to promote* rather than hinder *long-lasting quantum coherence in biological systems* (Plenio and Huelga, 2008; Huelga and Plenio, 2013). Because of the accumulating evidence that quantum phenomena need to be considered explicitly and in detail when studying living organisms, quantum biology has recently emerged as a new field at the border between quantum physics and the life sciences (Ball, 2011; Tarlac, 2011; Lambert et al., 2012; Al-Khalili and McFadden, 2014; Al-Khalili, 2017).

“Physicists thought the bustle of living cells would blot out quantum phenomena. Now they find that cells can nurture these phenomena—and exploit them” (Vedral, 2011).

So far, we have focused on non-trivial quantum processes in sensory cells. But are non-trivial quantum effects also present elsewhere in the nervous system? It is very likely, but direct experimental evidence is still missing. Where should we look for further instances of neuronal quantum effects? There are many stochastic neuronal mechanisms, which may be driven by quantum events. Although it is true that *“the main sources of neural noise are forces that can be characterized as thermal and chaotic rather than quantum in nature”* (Sompolinsky, 2005), quantum physics is expected to shape at least some stochastic events in the brain, such as the opening of ion channels (Vaziri and Plenio, 2010). In this way microscopic quantum events might affect electrical signals in neurons, as proposed by Paul Glimcher:

“[T]hese data suggest that membrane voltage is the product of interactions at the atomic level, many of which are governed by quantum physics and thus are truly indeterminate events. Because of the tiny scale at which these processes operate, interactions between action potentials and transmitter release as well as interactions between transmitter molecules and post-synaptic receptors may be, and indeed seem likely to be, fundamentally indeterminate” (Glimcher, 2005).

Johnjoe McFadden has made a similar suggestion:

“If neurons poised on the dynamics of individual membrane proteins are critical to the initiation of a particular course of motor action or cognitive process, then the consequent action or cognitive processes will be subject to non- deterministic quantum dynamics” (McFadden, 2002).

It is in itself important that quantum coherence in living organisms has been experimentally demonstrated at the microscopic level. But what are the spatial and temporal limits of these quantum effects? Can we discover quantum coherence in more than just a few molecules? How long can it persist? Some quantum brain proposals, focusing on explaining consciousness (Hameroff and Penrose, 2014), would require coherent quantum waves on much larger and longer scales than those found so far. The honest reaction to the questions just posed is that we do not know the answers to them and that only future research can provide those answers. The ‘orchestrated objective reduction’ (Orch-OR) hypothesis based on putative quantum computations in microtubules has been criticized for various reasons (Seife, 2000; Tegmark, 2000; McKemmish et al., 2009), e.g., for not being able to distinguish between neural mechanisms of conscious and unconscious information processing (Baars and Edelman, 2012). Although the authors of the Orch-OR model have tried to address most of the criticisms (Craddock et al., 2014; Hameroff and Penrose, 2014; Hameroff et al., 2014), their and other quantum consciousness hypotheses remain controversial and difficult to test experimentally. Interestingly, a new quantum cognition model, based on entangled calcium phosphate molecules in neurons, has recently been suggested and seems better testable than previous proposals (Fisher, 2015; Weingarten et al., 2016). One might speculate that future research on quantum cognition and consciousness might benefit from developing a quantum version of the integrated information theory (Tononi et al., 2016). Nevertheless, it is very important to emphasize that the main idea of this article, namely that biological evolution is able to take advantage of non-trivial quantum events at the microscopic level (see above) and even at larger-scale levels [by non-linear amplification, see point (2) below], is not dependent on the plausibility or validity of quantum consciousness proposals.

(2) If it turned out that quantum effects cannot be observed in living systems at the macroscopic level, would that mean that living systems can be fully described by classical physics? Or is there another plausible way in which small-scale quantum effects – there is evidence for their occurrence [see (1)] – might influence large-scale neuronal activity and behavior? Yes, there is. The common view that minuscule fluctuations, including quantum events, cancel out in larger systems need not be true in highly non-linear systems like our brain. The nervous system can be seen as a nested hierarchy of non-linear complex networks of molecules, cells, microcircuits, and brain regions. In iterative hierarchies with non-linear dynamics (at the edge of chaos), small (even infinitesimal) fluctuations are not averaged out, but can be amplified. Quantum fluctuations on the lowest level of scale may influence the initial state of the next level of scale, while the higher levels shape the boundary conditions of the lower ones. This hierarchy of nested networks with many feedback loops exploits rather than cancels out the quantum effects as proposed by Jeffrey Satinover:

“[Q]quantum dynamics alters the final outcomes of computation at all levels – not by producing classically impossible solutions but by having a profound effect on which of many possible solutions are actually selected” (Satinover, 2001).

In his essay on free will and neuroscience, Haim Sompolinsky has also mentioned this possibility:

“Chaos within the brain may amplify enormously the small quantum fluctuations … to a degree that will affect the timing of spikes in neurons” (Sompolinsky, 2005).

Similarly, even Christof Koch, one of the major critics of quantum brain ideas, had to admit:

“What cannot be ruled out is that tiny quantum fluctuations deep in the brain are amplified by deterministic chaos and will ultimately lead to behavioral choices” (Koch, 2009).

The quantum amplification mechanism has been adopted also by Scott Aaronson whose recent “freebit” theory of free will “postulates that chaotic dynamics in the brain can have the effect of amplifying freebits to macroscopic scale” (Aaronson, 2016, but see also criticism of this quantum ampflification idea in Clarke, 2014). A similar theory has already been proposed by Jordan (1938).

What is the evidence for the proposal that the brain is a complex non-linear system, capable of chaotic dynamics (van Vreeswijk and Sompolinsky, 1996; Harish and Hansel, 2015)? Beggs and Plenz (2003, 2004) provided experimental evidence that neuronal networks can produce complex patterns of collective activity, which are called *neuronal avalanches*. These avalanches have a characteristic distribution: each avalanche engages a variable number of neurons, but, on average, many more small avalanches are observed than large ones. This indicates that neuronal networks are poised *near criticality* (near phase transition, Beggs and Timme, 2012) and are prone to displaying emergent *complex* activity (Chialvo, 2010). Similar results supporting *criticality* in the brain have been obtained on a larger spatial scale from fMRI data (Deco and Jirsa, 2012) and from local field potential and spike recordings (Hahn et al., 2010, 2017 and references therein; but see also Touboul and Destexhe, 2010; Priesemann et al., 2014; Nonnenmacher et al., 2017). In general, we can observe three types of dynamics in the brain: (1) *ordered/subcritical* dynamics consisting of oscillatory synchronous activity with the characteristic features of high coordination and low variability, (2) *random/supracritical* dynamics consisting of asynchronous irregular activity with low coordination and high variability, and (3) *complex/critical* dynamics with high coordination and high variability. Brain states exhibiting *complex/critical* dynamics are the most interesting ones because they support the most efficient information processing (Beggs and Timme, 2012). At the critical point between order and disorder (i.e., at the edge of chaos and instability), neurons can communicate best, since at that point they are coordinated but not stuck in a certain state for a long time and can establish long-range dynamical correlations. Furthermore, neuronal networks in near critical states display, because of largest fluctuations, the largest repertoire of network activity. Finally, at the critical point, the highest sensitivity to small fluctuations (Fujisawa et al., 2006; London et al., 2010) is observed: even a single neuron perturbation has a small but non-zero chance to trigger an avalanche. As pointed out by Dante Chialvo, there are convincing Darwinian reasons for supposing that (parts of) our brains operate near the critical point (Chialvo, 2010): in a *subcritical* world, everything would always be uniform, there would be nothing new to learn and hence no critical and plastic brain would be needed; memories might as well be unchanging. In a *supracritical* world, everything would always be changing with no regularities to be learnt. No long-term plasticity and memory would be of any help. In our *critical* (complex) world, surprising events do occur, but regularities, too, are present so that the brain needs to register but also to update the stored memories.

“[B]rains seem “balanced on a knife-edge” between order and chaos: were they as orderly as a pendulum, they couldn’t support interesting behavior; were they as chaotic as the weather, they couldn’t support rationality” (Aaronson, 2016).

Thus, it is plausible, that small quantum fluctuations can be amplified, since brain activity can develop to the critical point: the point of complex neuronal dynamics. Interestingly, recent calculations suggest that quantum coherence can become long-lived in complex systems which are in a critical state between chaos and regularity – at the edge of quantum chaos (Vattay et al., 2014).

In summary, the intricate interplay between quantum effects and non-linear complex dynamics might be able (a) to generate new persistent quantum-chaotic patterns at a microscopic scale, (b) to amplify quantum effects to a macroscopic scale (Satinover, 2001). How exactly the indeterminacy of complex quantum dynamics of the brain is embedded in classical neuronal mechanisms of decision making (Rolls, 2012) remains to be determined.

## Toward Quantum Neurobiology?

How could the two scales (the microscopic and the macroscopic one) be interconnected? Is there a biologically plausible way in which small-scale quantum effects could influence large-scale neuronal activity and behavior? In their book, Al-Khalili and McFadden (2014) have made a specific proposal of such interconnection. According to their hypothesis, quantum events, e.g., at the level of ion channels, might affect and/or be affected by extracellular electric fields, which are generated at the level of neurons and their networks. This proposal is based on recent evidence that extracellular fields are able to influence membrane potential of neurons and their spiking activity by so called ephaptic coupling (Fröhlich and McCormick, 2010; Anastassiou et al., 2011; Anastassiou and Koch, 2015). There is evidence for a causal loop between brain’s endogenous electrical fields and neuronal firing mediated by voltage-gated ion channels. Electrical fields may guide and synchronize firing of many neurons and thus affect cognition and behavior. Thus it is possible that, by modulating (and being modulated by) ion channel activity, electrical fields are coupled to the level of quantum coherent events in many neurons, potentially affecting behaviorally relevant synchrony of neural firing (Al-Khalili and McFadden, 2014).

Interestingly, recent research in psychology has provided another hint that the brain displays quantum-like behavior (Bruza et al., 2015). This lead to the emergence of a new field of quantum cognition (Busemeyer and Wang, 2014). Several studies have shown that certain aspects of decision-making behavior are better described by quantum probability framework than classical probability framework (Mogiliansky et al., 2009; Pothos and Busemeyer, 2009; Yukalov and Sornette, 2011; Wang et al., 2014). However, the quantum cognition theory and its results do not necessarily imply that the brain employs quantum events to implement quantum cognition (Busemeyer et al., 2017). Whether quantum physics is related to the emergence of cognition from neural activity is still an open question (Bruza et al., 2015). Therefore, it will be exciting to see whether future experiments will find or disprove a link between ion channel coherence, field potentials and the above mentioned quantum-like decision behavior. Computational neuroscience may help design the appropriate experimental tests. Large-scale and highly detailed but still purely classical models of neurons and neural circuits are currently being developed (e.g., Markram et al., 2015). Quantum brain hypothesis predicts that these models will fail to account for some observed cognitive phenomena (such as quantum-like decision-making). It predicts that new mechanisms from quantum physics will have to be incorporated in these models. A more specific prediction of Al-Khalili and McFadden’s theory (see above) would be that successful models will have to include quantum coherence in ion channels as well as ephaptic coupling between endogenous electrical field potentials and cellular membrane potentials. Even if details of such quantum brain proposals will turn out to be incorrect, a thorough study of the possible contribution of quantum events to the computational aspects of the neural networks is worthwhile because it may lead to the discovery of novel principles of information processing in neuroscience as well as in artificial intelligence and neurorobotics.

## Learning from Non-Neuronal Biological Systems?

Long-lasting quantum coherence phenomena have been first observed in non-neuronal systems. Therefore, non-neuronal organisms such as bacteria, algae or land plants can be useful model systems to study the relationship between biological information processing and quantum events. Many molecular mechanisms, which support complex information processing, including neurotransmitter molecules, ion channels, gap junctions, evolved before the emergence of the nervous system during evolution (Baluska and Levin, 2016). Molecular and functional similarities between plants and neurons provide a hint that the notion of cognition defined as complex signal processing and signal integration, including perception, memory and decision-making, should be extended from neural to aneural systems (Baluska and Mancuso, 2009a; Baluska and Levin, 2016). Even though the concept of plant neurobiology (Brenner et al., 2006; Baluska and Mancuso, 2009b) has been controversially debated (Alpi et al., 2007; Brenner et al., 2007; Trewavas, 2007) and the essential requirements for conscious as compared to unconscious cognition remain the topic of investigation and discussion (Jedlicka, 2007; Trewavas and Baluska, 2011; Boly et al., 2017; Odegaard et al., 2017), an exploration of common principles underlying biological information processing in plants and animals leads to new insights and ideas. For instance, it has recently been suggested that both animals and plants are capable of active inference and anticipatory behavior (Calvo et al., 2016; Calvo and Friston, 2017). It is tempting to speculate that to achieve optimal accumulation of information (Jedlicka, 2007; Tkaćik and Bialek, 2014) and predictive coding, evolution would have developed strategies for exploiting the benefits of both classical as well as quantum information processing. Maybe our brain is not only a classical (Knill and Pouget, 2004; Friston, 2010) but, at least to some extent, also a quantum Bayesian inference machine (Haven and Khrennikov, 2016). Using simple organisms to better understand complex functions of the brain such as learning and memory has a great tradition in neuroscience (Kandel, 2001). Thus, studying bacteria or plants in the context of quantum biology (Lambert et al., 2012) may bring new insights also on quantum aspects of computations in the brain.

## Conclusion

Although new ideas and concepts are emerging (Van Regenmortel, 2004; Noble, 2010), reductionism and determinism are still the major paradigms in current biology, including neurobiology. *“[Physicists] invented the deterministic-reductionistic philosophy and taught it to the biologists, only to walk from it themselves”* (Loewenstein, 2013a). The dominant belief is that *“anything can be reduced to simple, obvious mechanical interactions. The cell is a machine. The animal is a machine. Man is a machine”* (Monod, 1974). Direct experimental evidence in favor of the quantum brain hypothesis would challenge the mainstream reductionistic-deterministic view of the human brain as a sophisticated machine performing classical computations.

## Author Contributions

The author confirms being the sole contributor of this work and approved it for publication.

## Conflict of Interest Statement

The author declares that the research was conducted in the absence of any commercial or financial relationships that could be construed as a potential conflict of interest.
